# Assessing the severity of thermal discomfort in a building in the course of hot and humid climate

**DOI:** 10.12688/f1000research.154075.1

**Published:** 2024-08-23

**Authors:** Thomas Janvier Matongo, Gilbert Roméo Hubert Ngock, Emmanuel Yamb, Léopold Mba, Benjamin Salomon Diboma, Jean Gaston Tamba

**Affiliations:** 1 Laboratory of Technologies and Applied Science, The University Institute of Technology, The University of Douala, Douala, Cameroon; 2 Department of Civil Engineering, Advanced Technical Teachers Training College, University of Douala, Douala, Cameroon; 3 Laboratory of Mechanics and Adapted Materials LAMMA, Advanced Technical Teachers Training College, University of Douala, Douala, Cameroon; 4 Higher Institute of Transport, Logistics and Commerce, University of Ebolowa, Ambam, Cameroon; 5 Laboratory of Transports and Applied Logistics, University Institute of Technology, University of Douala, Douala, Cameroon

**Keywords:** Residential building, Degree hour, Thermal discomfort, hot and humid weather, Douala.

## Abstract

This work is an application of experimental temperature data previously collected in a residential building in Douala, Cameroon, in order to analyze thermal discomfort. The data was collected according to three occupancy scenarios over 12 month period using thermohygrometer sensors. The temperature data are analysed in comparison with the comfortable temperature range from 24°C to 28°C. The degree hour (DH) method was used to assess the severity of thermal discomfort in a hot and humid climate. The results reveal that the open C1, closed C2 and inhabited C3 rooms corresponding to scenarios C1, C2 and C3 respectively, have 7270.6°H, 9063.9°H and 10023°H. The inhabited room C3 has the largest DH and although the room C1 has the smallest DH, the latter largely exceeded the tolerable limit value of 1250°H set by the RE2020 Environmental Regulations. Results from this work can serve in building modelling for researchers and architects to act for the alleviation of thermal discomfort in regions with hot and humid climate.

## 1. Introduction

Douala is a coastal city located 35km from the Gulf of Guinea in Cameroon. The city is characterized by a hot and humid weather regularly crossed by light winds of 3 to 7 km/h. Demographically and according to the National Institute of Statistics (INS) in 2019, Douala has more than 3 million inhabitants. The most urbanized area of the city is 210 km ^2^ with 70 km ^2^, 70% of which is built according to the Douala Urban Community (CUD). The demographic boom and the rural exodus have favoured an increase in demand for housing, leading to anarchic occupation of land and non-compliance with architectural rules adapted to the climate. This situation has led to an increase in heat islands in the city. ^1^^,^^2^ Cinder block constructions abound due to the availability of this material on the market. The first concrete houses in the region met certain provisions and criteria such as the orientation of doors and windows in relation to the prevailing wind, ventilation holes, shutters, and prefabricated screens on the upper parts of the bays. This fosters regular home ventilation even when the bays are closed. Nowadays, these criteria are largely neglected. The trend towards glass doors and windows predominates. We regretfully note that the habits of the occupants of modern houses are among the causes of discomfort since the openings are sometimes not used properly. Doors and windows remain constantly closed, which does not favour wind circulation and air renewal. Studies related to indoor air quality show that poor air renewal can cause many illnesses, such as allergies, asthma, lung infections, headaches, as well as nose, throat and eyes irritation. Furthermore, better thermal comfort can be obtained with a good design of habitats that are adapted to the climate of the city of Douala. ^3^ In 2012, some studies on thermal comfort conditions in the city resulted in the temperature range of 24°C to 28°C and for an interval of 60% to 69% relative humidity. ^4^^,^^5^

However, thermal discomfort in buildings has become a major nuisance in Douala, ^1^ especially for modern housing made from unconditioned cinder block. The intensity of this problem which has so far been little assessed is observed at different degrees in the rooms of the same building. These differences are mainly related to the behavior of the inhabitants, wind circulation, air temperature and humidity. Oluwafemi et al in 2010 ^6^carried a study in houses in Bauchi in Nigeria, and showed that thermal comfort depends on the adaptation of the occupants in their residence. It has been demonstrated that despite the existence of several factors acting on the interior environment, the occupant seems to be the most impactful element. In fact, the action of the occupant on the window and the shutter modifies air renewal as well as the management of solar gain. The works of Alison and Chungyoon in 2003 ^7^ demonstrated that occupants of well-ventilated rooms have a good thermal sensation.

To assess thermal comfort, certain studies have particularly used the PMV (Predicted Mean Vote) method. ^4^^,^^5^^,^^7^ Which aims to predict the mean value of a group of occupants on a seven point thermal sensation scale.ai Her evaluation being subjective. In addition, when it is necessary to express the severity of discomfort by period, methods such as PMV, PPD (Predicted Percentage of Dissatisfied) fail to meet this objective, and this limits their usefulness in many cases. ^8^ On the other hand, the DH method offers the possibility of assessing the severity of discomfort by temporal stages in an inhabited or uninhabited building, which justifies its choice for the purpose of this study.

Several studies carried in recent decades have used the DH as an index for assessing discomfort. In 2010, studies revealed the influence of exterior shading systems for a substantial reduction in DH at the level of intermediate floors during cooling periods. ^9^ In 2013, a study carried out in a well-insulated commercial building, located in a temperate climate, showed the benefit of natural night-time ventilation provided by opening the skylights as a passive cooling strategy, which contributes to a significant reduction in DH by approximately 19% for the entire building in summer. ^10^ In 2018, studies carried in four Moroccan climatic areas revealed that the thermal comfort of occupants increases in rooms with cantilevered beams, and the DH decreases by approximately 2.1% compared to rooms without cantilevered beams. ^11^ Nisrine et al in 2021 studied DH in a public building whose envelope consists of four facades, the ceiling and a roof. ^12^ They showed that the number of occupants strongly affects the DH whose intensity varies in the same direction as the number of occupants. Other studies have shown the importance of the DH index in building design since it can be used to evaluate thermal comfort objectively in buildings in inhabited or uninhabited mode. ^8^^,^^12^ The RE2020 environmental regulation in France defines two DH thresholds: A 350°H threshold below which the building is comfortable, and a 1250°H threshold above which the building shows an unacceptable discomfort. Between these two thresholds we talk about “tolerable discomfort”. ^13^ Thus, DH studies in the South Sahelian African area should be promoted within the framework of local regulations.

From the literature review, it appears that the level of thermal discomfort is a primary factor in assessing building performance. Also, the DH index is one of the evaluation methods often used for a specific occupancy scenario in a building. The originality of this work lies in the fact that thermal discomfort is assessed in the same building according to three simultaneous occupancy scenarios representing the real situations of a habitat. We wonder about the intensity of DH in a room depending on whether it is inhabited, closed uninhabited or open uninhabited.

The purpose of this work is to assess the intensity of thermal discomfort based on experimental data and using the DH index in a hollow agglomerate block room located in a hot and humid area, following three simultaneous scenarios with data previously collected over a12 month period.

The methodology adopted in this work begins with the construction of an experimental building of R+1 with three test rooms of the same measurements upstairs. The data acquisition system which consists of thermohygrometers and a computer is installed in the experimental premises. Three occupancy scenarios are then defined for the three test premises: the first premises is open and not occupied during the experimentation phase; the second is closed and not occupied and the third is occupied in real conditions according to the behaviour of people in the region. The collection, processing and storage of air temperature and relative humidity data from the different rooms is carried out. Moreover, the thermal discomfort index is determined by the method of the degree of discomfort hours of the premises and the thermal discomfort rate.

## 2. Methods

### 2.1 Geographical location and specific climate conditions of the area of study

**Figure 1.**
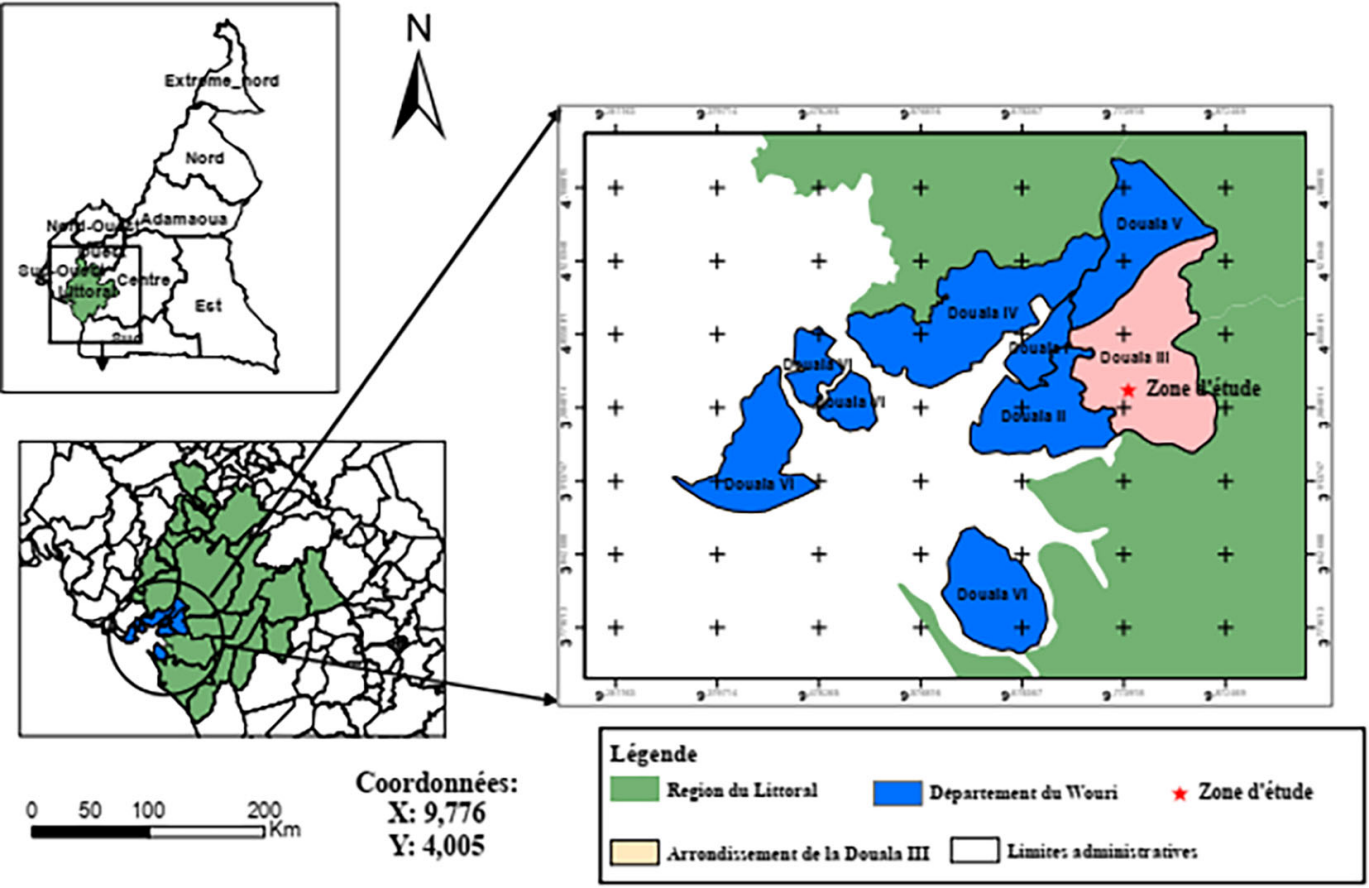
Study region. This figure was reproduced with permission from the autors. ^20^

The experiment was carried out in Douala which is a city located in the Littoral region of Cameroon, a country in central Africa. Cameroon stretches from the Gulf of Guinea to Lake Chad. It extends in length from the 2nd to 13th degree of North latitude and in width from 6th to 16th degree of East longitude. The opening to the sea and its stretch give the country a wide variety of climates. ^14^^,^^15^ The Littoral region is positioned from the 3°57’30”N to 4°7’30”N and from the 9°37’30”E to 9°50’0”E ( Figure 1). The place is an area with a hot and humid climate. ^5^^,^^16^ Humidity in this area varies between 65% to 98% and temperatures between 19°C and 32°C with an average of 25°C. ^17^ Douala records high rainfall due to the proximity of the city to the Atlantic Ocean, that is 175 to 200 rainy days per year, with a water depth of 4500mm. ^18^ The most urbanized area is 210 km ^2^ and according to INS, littoral statistical yearbook 2019, Douala had 3,049,034 inhabitants in 2018 with an average annual population growth rate estimated at 5% per year over the last 30 years. ^19^

### 2.2. Description of the experimental building

**Figure 2.**
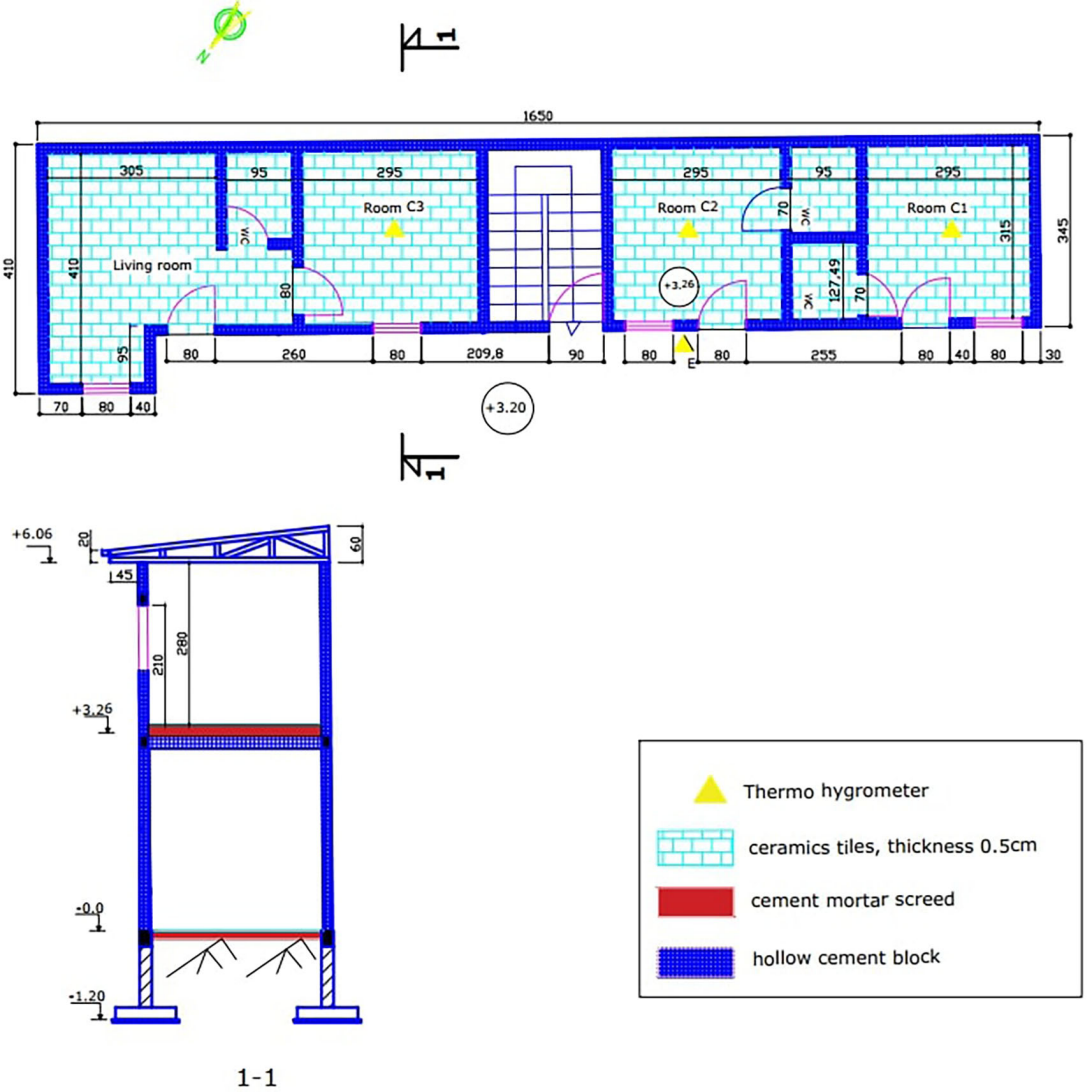
Building plan and cross section.

The experimental building is a modern R+1 building with cement blocks as an envelope. The building has three rooms of the same size, built upstairs. The three (03) experimental rooms have equivalent surfaces of 9.30m2. The walls are built from 15x20x40cm cinder block. The thickness of the cement mortar coating is 2cm. The interior and exterior surfaces of the walls are painted white. The floor is a 16x20x50cm hollow body floor covered with a 6cm thick screed and 0.5cm thick ceramic tiles. The roof is made of 0.5mm aluminum sheets, with a 4mm thick plywood ceiling. There are wooden doors measuring 3x80x210cm. We also have glazed windows of 0.4x80x100cm. The ceiling is 2.80m high ( Figure 2). There is data acquisition equipment in the experimental rooms and in the outdoor environment.

For this purpose, three occupancy scenarios were carried out

- Scenario 1 (C1): open uninhabited room with the door and windows open;
- Scenario 2 (C2): closed uninhabited room with the door and window closed;
- Scenario 3 (C3): inhabited room.

### 2.3. Thermohygrometer

It is a temperature and relative humidity recorder, manufacturer by EXTECH®, RHT10 model; **Copyright** © **2014 FLIR Systems, Inc.** These characteristics are given in Table 1 below.

**Table 1.** Characteristics of the thermohygrometer.

Relative Humidity (%)	General amplitude	0 ⇒ 100
	Accuracy (0 ⇒ 20 and 80 ⇒ 100)	± 5.0
	Accuracy (20 ⇒ 40 and 60 ⇒ 80)	± 3.5
	Accuracy (40 ⇒ 60)	±3.0
Temperature (°C)	General amplitude	- 40 à 70
	Accuracy (-40 ⇒ -10 and + 40 ⇒ + 70)	± 2
	Accuracy (-10 ⇒ 40)	±1
Download rate	Selectable sampling interval: from 2 seconds to 24 hours	
Operating Temperature (°C)	-35 ⇒ 80	

### 2.4. The computer

A laptop computer Intel® core ™ i5-4200U CPU @ 1.60GHz 2.30 GHz, is used for processing and drawings

### 2.5. Data acquisition

Thermohygrometers are suspended from the ceiling, 1.10 m from the floor inside each of the three rooms (C1, C2 and C3), in accordance with ASHRAE standard 55. Another thermohygrometer is placed in the exterior environment to acquire the temperature and humidity of the outdoor air. The data recording interval is set at 1 hour. Temporal data is collected over a one-year period, from January 2019 to December 2019.

### 2.6. Thermal discomfort assessment method

To assess discomfort, we used the hour degree method. Then, we calculated the discomfort rate.

**2.6.1. DH (Degree of summer discomfort time)**

The DH is the sum of the positive differences between the interior temperature and the constant threshold temperature common to all building uses. It is determined over a one-year period and expresses the duration and intensity of periods of discomfort in the building. ^9^^–^^11^^,^^21^^–^^25^ His makes it possible to calculate the sum of discomfort intensities per year in a room, ^26^^,^^27^ hour by hour see equation 1. ^23^^,^^25^^,^^28^

$$\mathrm{DH}=\sum_{i=1}^{N}(T_{i}-T_{s})\;(\mathrm{si}\;T_{i}>T_{s}).$$ (1)

**Ti** = Resulting interior temperature.

**Ts** = Comfort threshold air temperature and has a value of 28°C. ^4^^,^^5^

**DH** = Degree hour.

**N** = Total number of hours over a year which is 8760.

According to the RE2020 environmental regulation, there are two thresholds for DH. ^8^^,^^27^

• 1 low threshold of 350°H => Comfortable building
• Between 350°H and 1250°H => Discomfort is tolerable
• 1 high threshold of 1250°H => Uncomfortable building

**2.6.2. Summer discomfort rate**

To determine the proportion of annual hours of discomfort in each room, we calculated the discomfort rate ( equation 2). ^11^

$$\mathrm{Hot}\;\text{discomfort rate}=\frac{\text{Number of hours T}>28^{{}^{\circ}}C}{8760}.$$ (2)

## 3. Results and Discussions

Data collected outside and in the experimental premises are analyzed, and the thermal comfort in the premises is assessed by season and over the entire year.

### 3.1 Analysis of outdoor environment data

As in other previous studies, the monthly average temperature and humidity data in Table 2 show that Douala has a hot and humid climate. ^17^^–^^18^^,^^20^ February is the hottest month with an average temperature of 30.8°C ±2°C, while August is the coldest with an average temperature of 26.4°C ±2°C The average humidity varies between 75% and 87.7% ±8%. ^29^

**Table 2.** Average thermohygrometric data of the outdoor environment.

Months of the year	1	2	3	4	5	6	7	8	9	10	11	12
Average Temperature ±2°C	29.8	30.8	29.9	30.3	30.1	28.4	27.2	26.4	27.8	28.0	28.6	30.2
Average Hygrometry ±8%	77.8	75.7	76.3	75.8	75.0	81.2	85.5	87.7	83.3	82.1	80.5	76.8

### 3.2. Analysis of data from indoor environments of premises

The temperature data are analysed in comparison with the comfortable temperature range from 24°C to 28°C

**3.2.1. Analysis during the hot season**

**Figure 3.**
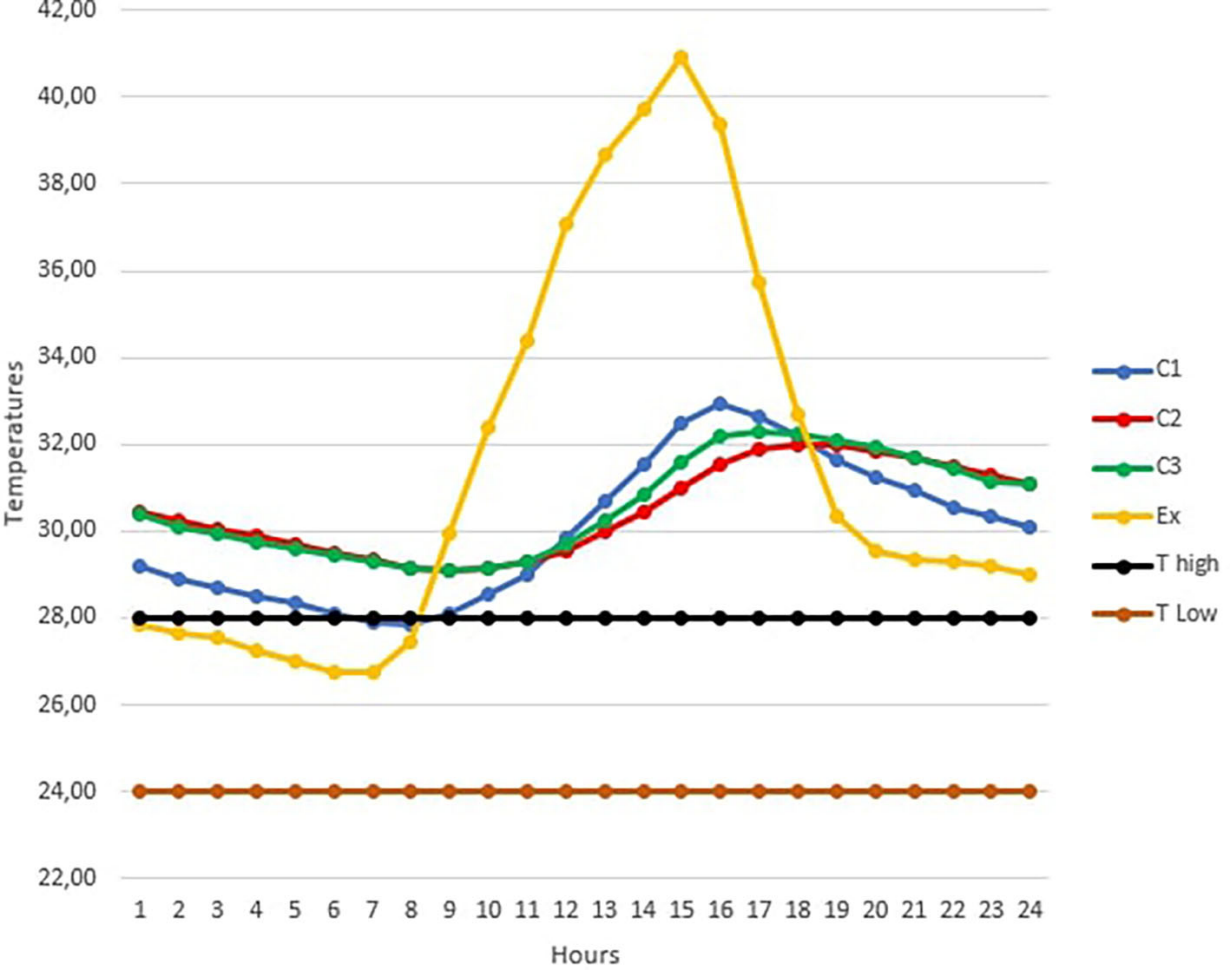
Temperature curves on the hottest day compared with the upper and lower comfort limit temperatures (24°C-28°C).

**Figure 4.**
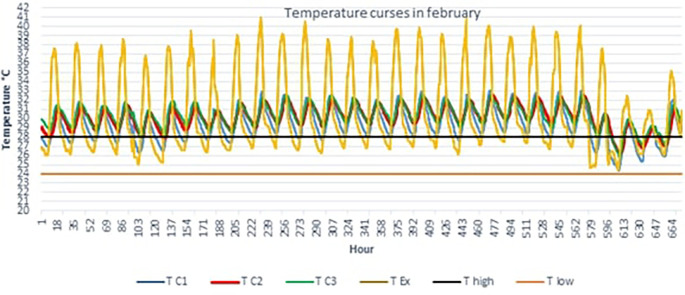
Temperature curves in the hottest month compared with the upper and lower comfort limit temperatures (24°C-28°C).

a) **The hottest day**
Figure 3 below shows the TC1, TC2, TC3 and TExt graphs of the hottest day in February over 24 hours.
  The maximum outdoor temperature was reached on February 10 at 3 p.m. with 40.93°C (RH=43.8%).
  Looking at Figure 3, we see that this day was particularly marked by high temperatures above the upper threshold temperature for thermal comfort in the three occupancy scenarios.
  For the open room C1, the heat peak was reached at 4 p.m. with 32.93°C, while the peak was observed at 6 p.m. in the closed room C2 with 32°C. Finally, the peak is reached at 5 p.m. in the occupied room C3 with 32.27°C.
  The curves of rooms C2 and C3 are almost identical over certain time intervals. This can be explained by the absence of the occupant in the room at these times.
b) **The hottest month**
Figure 4 below shows the TC1, TC2, TC3 and TEx graphs over 28 days for the month of February.
  Outdoor temperatures (TEx) vary from 25 to 41°C, the peak of outdoor temperatures is generally reached at 3 p.m. The temperature peak in rooms C2 and C3 is observed around 6 p.m., i.e. a peak phase shift of 3 hours compared to the outside. This can be explained by the inertia of the building envelope. We see that the temperature curves TC2 and TC3 of scenarios C2 and C3 are above the maximum threshold temperature (T high) from February 1 to 25. For scenario C1, we observe small, comfortable periods of 2 to 3 hours early in the morning.

**3.2.2. Analysis during the cold season**

**Figure 5.**
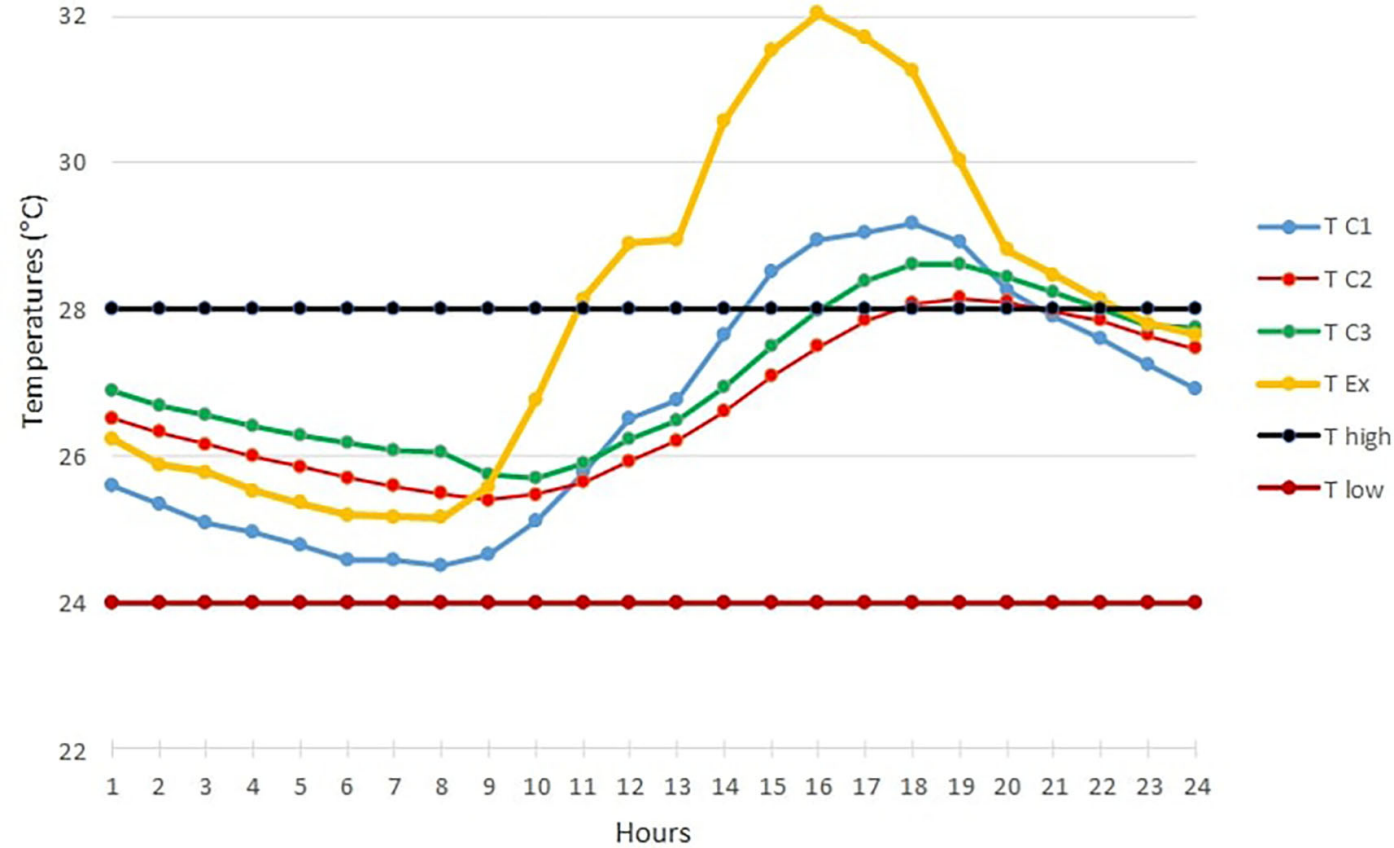
Temperature curves on the coldest day compared with the upper and lower comfort limit temperatures (24°C-28°C).

**Figure 6.**
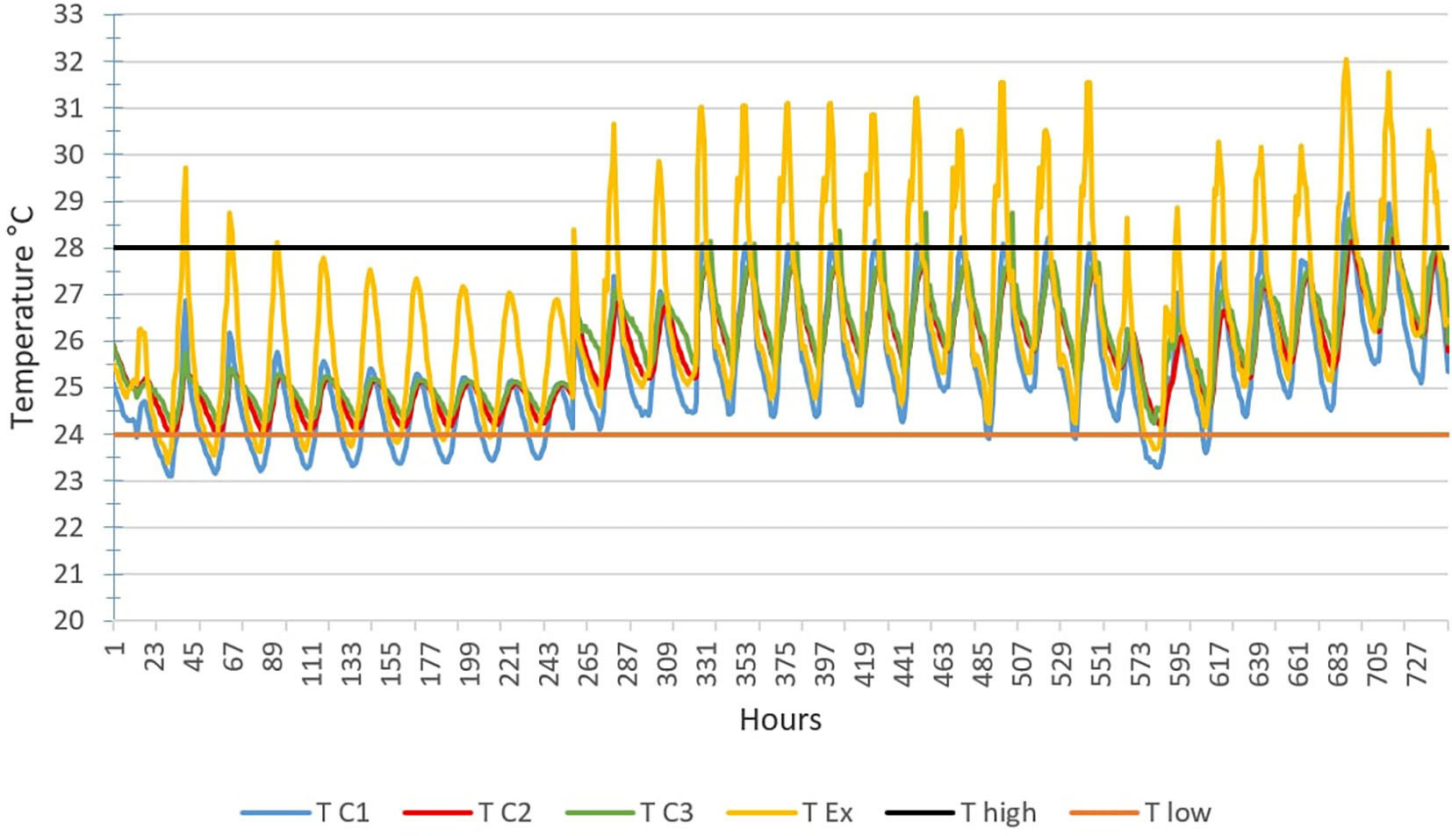
Temperature curves for the coldest month compared with the upper and lower comfort limit temperatures (24°C-28°C).

a) **The coldest day**
Figure 5 below shows the TC1, TC2, TC3 and TEx graphs over 24 hours of the coldest day of the month of August.
  The minimum outdoor temperature was obtained on August 29 at 4 p.m. with 32.04°C (Rh = 69.6%).
Figure 5 shows the temperature progress in the rooms and outside compared to the hot comfort threshold. It is noted that the heat peak was reached at 6 p.m. for the three occupancy scenarios with 29.17°C for C1, 28.08°C for C2 and 28.61°C for C3.
  It is a particularly comfortable day in the three rooms with a period of discomfort ranging from 3 p.m. to 9 p.m. for room C1, from 4 p.m. to 10 p.m. for room C2 and from 6 p.m. to 9 p.m. for room C3.
b) **The coldest month**
Figure 6 below shows the TC1, TC2, TC3 and TEx graphs over 31 days for the month of August.
  Outdoor temperatures (TEx) vary from 23°C to 32°C, the peak of outdoor temperatures is generally reached at 3 p.m. The temperature peak in rooms C2 and C3 is observed around 6 p.m. We see that the temperature curves TC2 and TC3 remain between the two high and low thresholds from August 1 to 28. For scenario C1, we observe periods of exceeding the lower limit of thermal comfort of 2 to 7 hours during the first 10 days of the month. This can be explained by the light rains that fall daily during this period.

**3.2.3 Analysis during the entire year**

**Figure 7.**
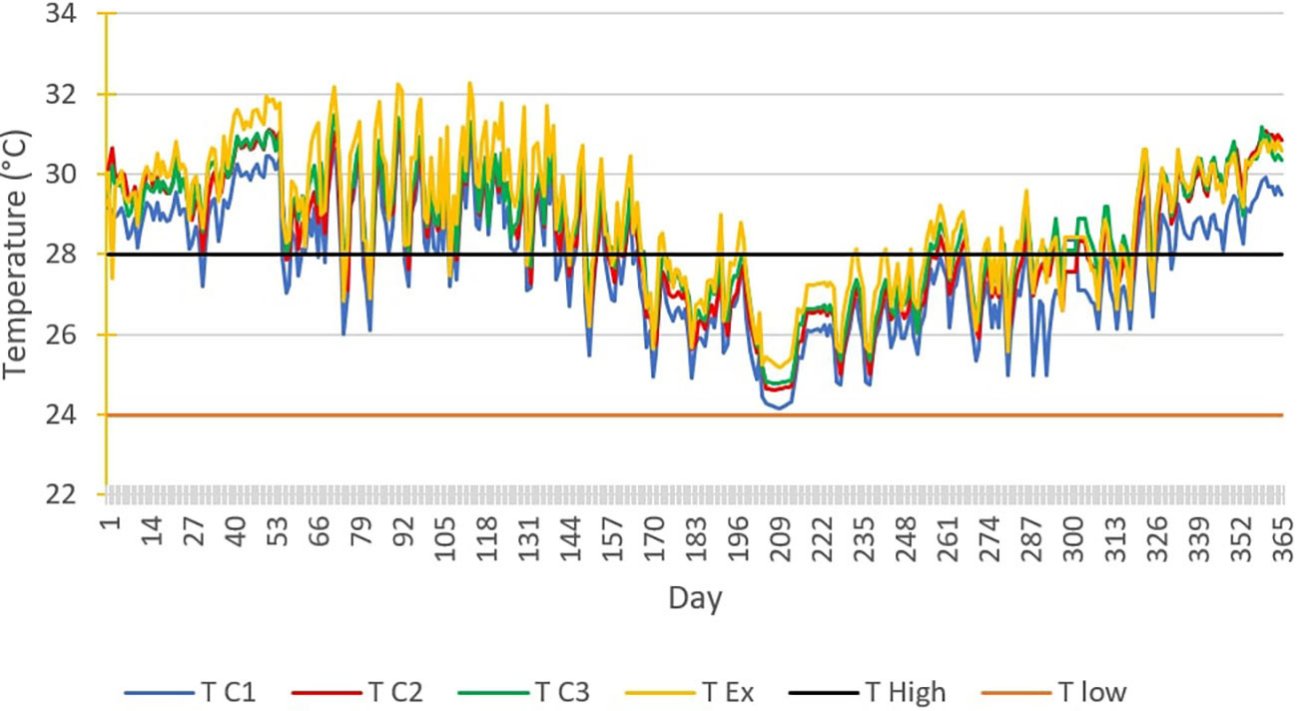
Temperature curves for 365 days of the year, compared with the upper and lower comfort limit temperatures (24°C-28°C).

Figure 7 below shows the TC1, TC2, TC3 and TEx graphs over the 365 days of 2019.

Thermal discomfort is noted in closed rooms C2 and inhabited rooms C3 on one hand, extending over a large number of days, except between the 200th and 260th day of the year when acceptable thermal comfort can be observed. On the other hand, in the uninhabited open room, a long period of acceptable comfort is observed between the 179th and 325th day. These periods of acceptable thermal comfort correspond to the main rainy season.

### 3.3. Indicators of thermal discomfort

The degrees of discomfort hours and the thermal discomfort rate in the test rooms for the three scenarios are shown in the figures below over the year.

**3.3.1. The number Degree hours of thermal discomfort**

**Figure 8.**
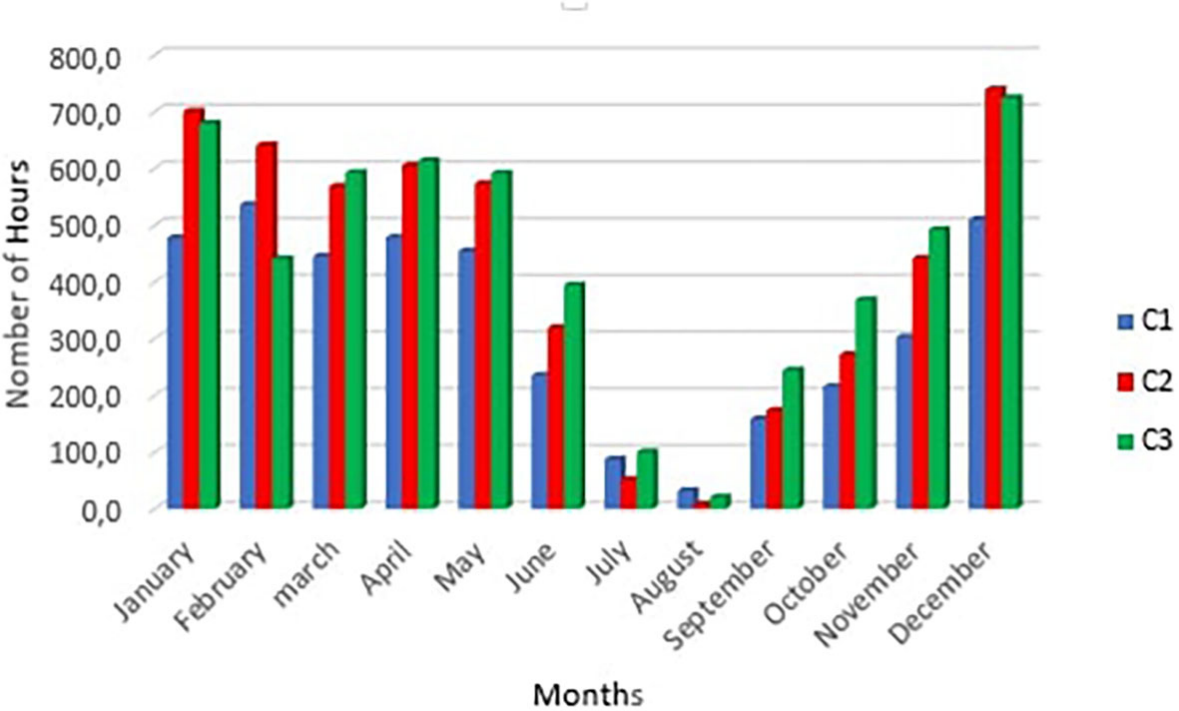
The number of hours of summer discomfort in rooms.

Figure 8 below shows the number of summer discomfort hours per month for scenarios C1, C2 and C3

We notice a peak in hours of discomfort in the month of December for scenarios C2 and C3 with respectively 739 hours and 724 hours, on one hand. ON the other hand, for scenario C1 the peak is reached in February with 536 hours this can be explained by the drop humidity in February. Furthermore, there was a considerable drop in the number of hours of discomfort in July and August. This period corresponds to the rainy season with fine drops falling throughout the day making the air humidity very high. The greatest number of hours of discomfort occurs in December in rooms C2 and C3. This may be due to fog despite the high temperatures.

**3.3.2. Degree hour of thermal discomfort**

**Figure 9.**
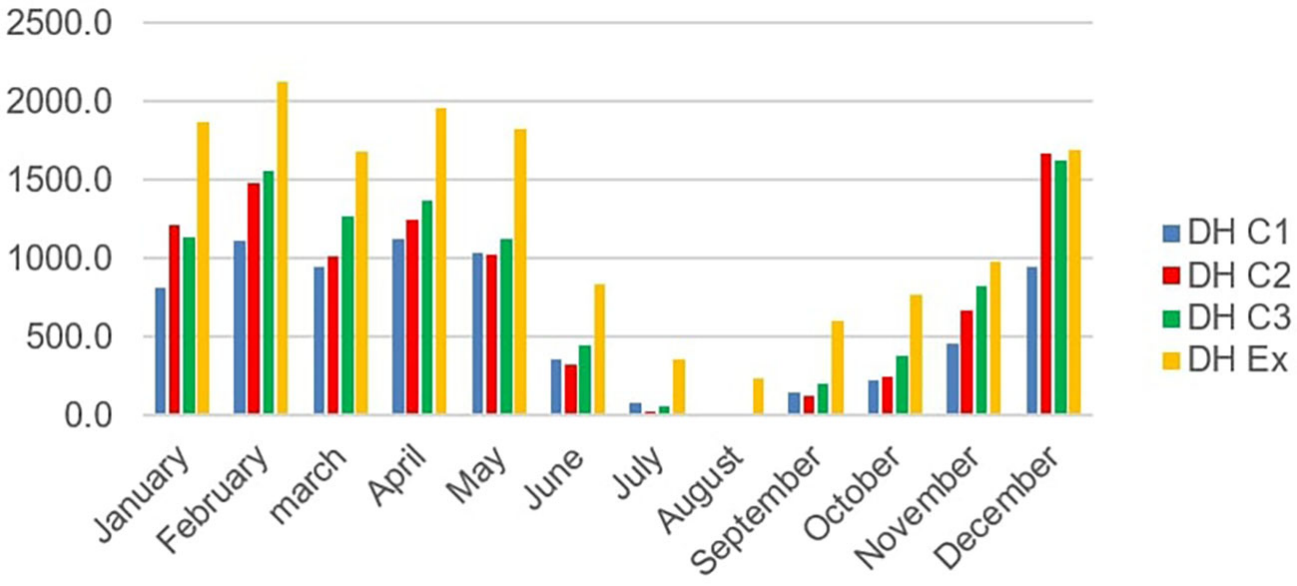
Monthly hours of rooms.

**Figure 10.**
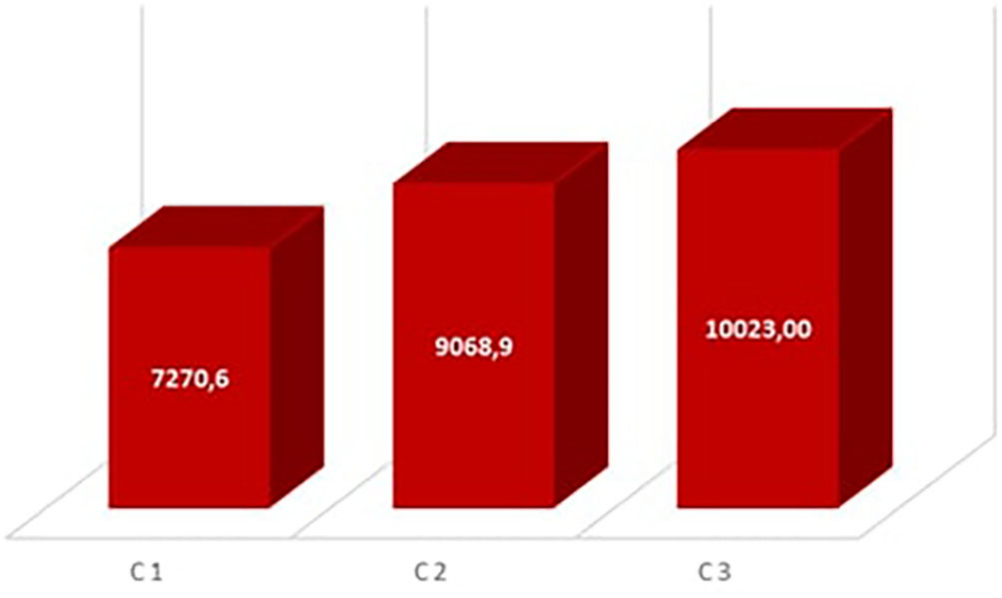
The Degree hours of the annual discomfort in the rooms.

a) **Degree hour of monthly summer discomfort**
Figure 9 shows the degree hours of monthly discomfort for scenarios C1, C2 and C3.
  A decrease in the DH is observed during the rainy season which extends from July to October as well as a period of tolerable discomfort in July and August with less than 100°H/month.
b) **The Degree hour of the annual discomfort**
Figure 10 shows the degree hours of annual discomfort for the occupancy scenarios. Room C3 has the highest discomfort degree hour with 10023°H, followed by room C2 with 9063.9°H, while room C1 has 7270.6°H. This corroborates the results of other research works such as that of Nisrine Laghmich et al. in 2021 ^15^ according to which the DH increases in the same direction as the number of occupants. Results also reveal that the renewal of air linked to the opening of the bays leads to an annual reduction in DH.

**3.3.3. The Degree hours of summer thermal discomfort of the three scenarios compared to RE2020**

**Figure 11.**
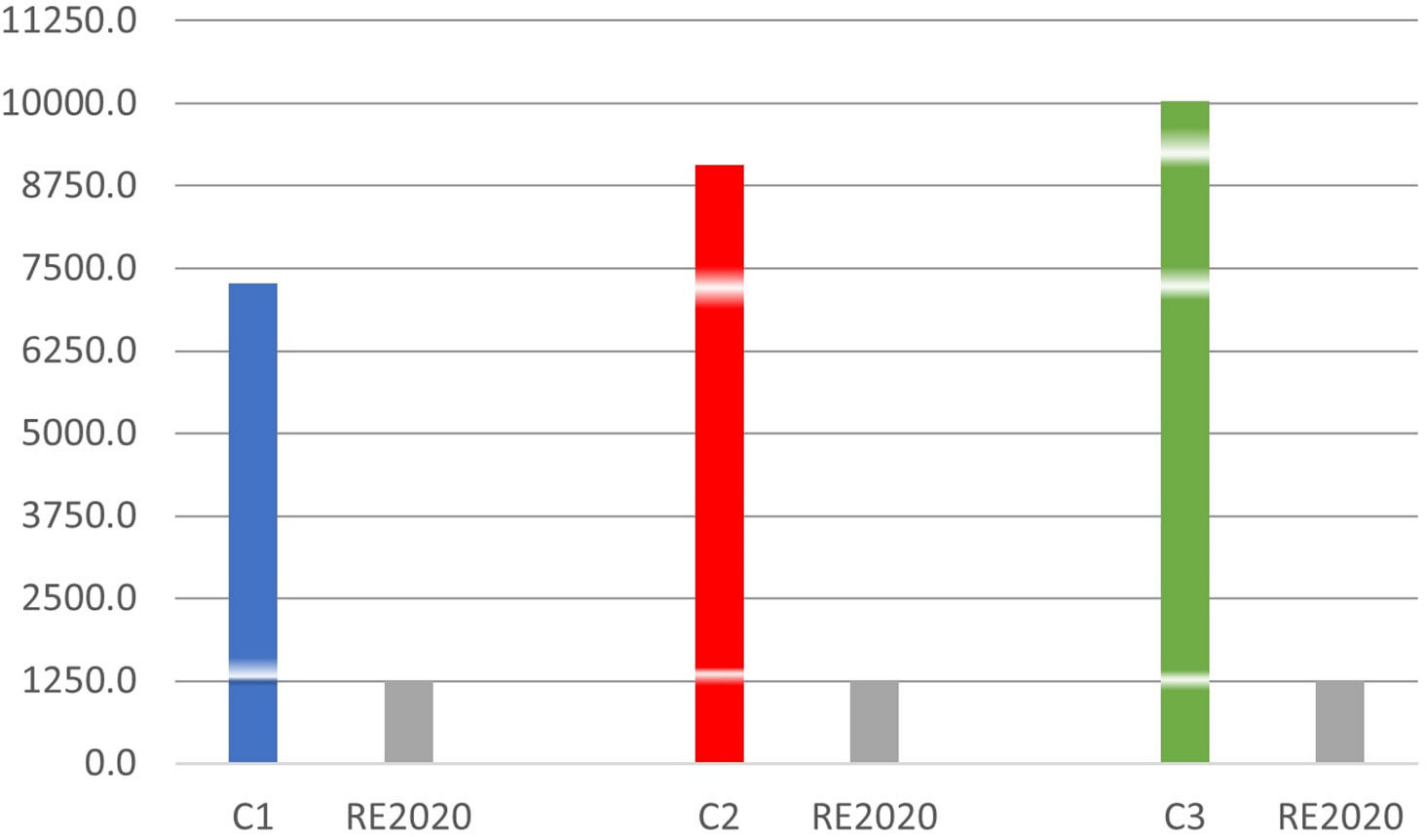
Degree hours of rooms compared to RE2020.

Compared to RE2020 which sets the maximum DH at 1250°H, ^17^ Figure 11 above shows that:

The DH is 5.8 times higher in room C1, 7.2 times higher in room C2 and 8 times higher in room C3. We notice that the discomfort in a premises made of concrete blocks in a hot humid climate is far above the tolerable limit value provided for by RE2020. Although these are unlivable conditions in other countries, the inhabitants of the city of Douala seem to adapt well. ^4^

### 3.4. The summer thermal discomfort rate

Table 3 below gives the percentage of annual hours where the temperature is above 28°C.

**Table 3.** Scenario discomfort rate.

Scenarios	C1	C2	C3
Discomfort rate	44.9%	58.1%	60.0%

Table 3 shows that:

• The discomfort rate in the closed and uninhabited room C2 is relatively low compared to the inhabited room C3. This can be explained by human presence which is a source of heat. ^30^
• For the open room C1, the discomfort rate decreases by 19.8% compared to the closed room. This can be explained by the opening of the windows which promote permanent ventilation of the room. Other studies in temperate climates have shown that natural night time ventilation provided by the opening of skylights contributes to a 19% reduction in DH in summer discomfort. ^31^

## 4. Conclusion

This work allowed to assess the severity of summer discomfort in a residential building made of cinder block in the city of Douala, in Cameroon. We have set to study the variation in the degree of discomfort time following different scenarios in three rooms of the same measurements. Scenario 1 (C1) with an open uninhabited room with the doors and windows open, Scenario 2 (C2) with a closed uninhabited room with doors and windows closed, and Scenario 3 (C3) with an inhabited room. The results obtained show that the DH in a room with a hollow agglomerate block envelope in a hot and humid climate exceeds more than five times the tolerable limit of 1250°H set by RE2020. Opening doors and windows allows a reduction in DH of 19.8% compared to a closed room. Occupants have a strong impact on the increase in DH due to the fact that they are a source of heat and also due to their action on the bays. Nevertheless, opening doors and windows does not seem to be enough to make the building comfortable. The observation made in this work can be remedied by solutions that are applicable to buildings in this type of climate. Namely, a better choice of construction site, good orientation of the building and openings, good organization of spaces, good protection of the building against solar rays, good natural ventilation and a reasonable choice of construction materials. For that purpose, this study proves to be useful to designers and real estate developers in order to make an optimal choice with regard to the building envelope, its protection against solar radiation and natural ventilation. It can also be important that room occupants take action to renew the air and reduce solar radiation. Eventually, the study can help public authorities improve regulations in the real estate sector in hot and humid climates.

## Data Availability

The data used in this work are available online in a Data Article: **Experimental data showing the thermal behaviour of a residential building in a hot and humid climate on three scenarios: An empty room with a closed door, an empty room with an open door, and a normal inhabited room**, doi: https://doi.org/10.1016/j.dib.2022.107906. ^29^ That data article contains the following underlying data under the terms of the CC BY license ( http://creativecommons.org/licenses/by/4.0)
1- tables of raw data collected in the study area over a 12-month period 2- figures (photos and curves)
