# Supplementary material for: Assessing the severity of thermal discomfort in a building in the course of hot and humid climate
Source: F1000Res. 2024 Nov 27;13:962. Originally published 2024 Aug 23. [Version 2] doi: 10.12688/f1000research.154075.2 (PMC11624430; doi:10.12688/f1000research.154075.2)
Supplement: Peer review history

# Reviewer report: Reviewer response for version 2

**DOI:** 10.5256/f1000research.175123.r343910

**Published:** 2024-12-06

**Authors:** Klodjan Xhexhi

**Affiliations:** 1 Polis University, Tiranë, Albania

According to my expertise the article is **Approved **to be indexing. My concerns are better exposed. Thank you for your consideration.

Is the case presented with sufficient detail to be useful for teaching or other practitioners?

Partly

Is the work clearly and accurately presented and does it cite the current literature?

Yes

If applicable, is the statistical analysis and its interpretation appropriate?

Yes

Are all the source data underlying the results available to ensure full reproducibility?

Yes

Are the conclusions drawn adequately supported by the results?

Yes

Is the background of the case’s history and progression described in sufficient detail?

Yes

Reviewer Expertise:

Bioclimatic Architecture; Building Materials; Architecture Technology; Building Energy Efficiency

I confirm that I have read this submission and believe that I have an appropriate level of expertise to confirm that it is of an acceptable scientific standard.

# Reviewer report: Reviewer response for version 1

**DOI:** 10.5256/f1000research.169057.r321444

**Published:** 2024-09-24

**Authors:** Klodjan Xhexhi

**Affiliations:** 1 Polis University, Tiranë, Albania

**General Comments**

**Relevance and Contribution**:

 The study addresses an important issue related to thermal discomfort in residential buildings in hot and humid climates, with a focus on the city of Douala, Cameroon.

 The use of the Degree Hour (DH) method is appropriate and relevant for quantifying thermal discomfort. This provides valuable information for architects and policymakers.

**Clarity and Structure**:

 The paper is well-structured, moving logically from introduction to methods, results, and recommendations.

 The background and rationale for the study are adequately explained, and the methodology is presented in sufficient detail.

**Strengths**

 The long-term data collection over 12 months enhances the reliability of the findings.

 The use of three distinct occupancy scenarios (open, closed, and inhabited rooms) provides a comprehensive analysis of different real-world conditions.

**Methodological Approach**:

 The DH method is effectively used to quantify thermal discomfort, and the study provides a clear metric for assessing discomfort in residential buildings.

**Recommendations and Practical Implications**:

 The paper offers practical recommendations for building design in hot and humid climates. These include considerations for natural ventilation, building orientation, and material choices.

**Weaknesses**

 The method adopted for DH is adequate, though perhaps not widely known by all readers. Further explanation is needed, or at least a short description of the formula and how it is applied in this work. How about DH norms in your country?

 (If they do exist).

 It would be further elaboration to understand how the analysis of the different parameters involved, such as humidity and occupants' behavior, influences DH values in each scenario.

 How the modern technologies of cooling or new building materials can offer further relief from thermal discomfort.

**Statistical Analysis:**

 In the context of this paper, which focuses on DH values, there are no statistical tests about the significance of the differences among scenarios. Having this would increase the weight of the conclusions.

**Literature Review**

 The literature review is adequate but would benefit from the addition of more recent works on thermal comfort and building design in similar climates-particularly those offering novel solutions.

**Conclusion and Implications:**

 The conclusions are generally supported by the results, but they could be more specific with policy implications and recommendations for further research.

**Recommendations for Improvement**

  -A brief non-technical description of DH method will be useful to readers who are unfamiliar with the metric.

 -All statistical tests shall be performed to see significance of differences between various scenarios.

 -Widen literature review by including more recent studies and references, especially dealing with advanced building technologies and materials.

 -Elaborate on specific policy recommendations as well as practical applications for architects and builders.

 -Please do not use the word “we observe” (for instance) instead of “It is observed that”. This is valid for the entire text.

 Although these facts are a good starting point for understanding thermal discomfort in residential buildings within hot and humid climates, a more developed analysis and a broader context would strengthen the article.

Is the case presented with sufficient detail to be useful for teaching or other practitioners?

Partly

Is the work clearly and accurately presented and does it cite the current literature?

Yes

If applicable, is the statistical analysis and its interpretation appropriate?

Yes

Are all the source data underlying the results available to ensure full reproducibility?

Yes

Are the conclusions drawn adequately supported by the results?

Yes

Is the background of the case’s history and progression described in sufficient detail?

Yes

Reviewer Expertise:

Bioclimatic Architecture; Building Materials; Architecture Technology; Building Energy Efficiency

I confirm that I have read this submission and believe that I have an appropriate level of expertise to confirm that it is of an acceptable scientific standard, however I have significant reservations, as outlined above.

## References

[1] : Assessing the severity of thermal discomfort in a building in the course of hot and humid climate. *F1000Research* .2024;13: 10.12688/f1000research.154075.1 10.12688/f1000research.154075.1 PMC1162443039649838

# Reviewer report: Reviewer response for version 1

**DOI:** 10.5256/f1000research.169057.r321441

**Published:** 2024-09-23

**Authors:** Khalid El Harrouni

**Affiliations:** 1 Ecole Nationale d'Architecture de Rabat, Rabat, Morocco

The paper uses experimental data to assess and quantify thermal discomfort in residential buildings in a hot and humid environment. Its reliance on the DH method provides a clear metric for thermal discomfort, and its focus on real-world applications makes it relevant for architects, engineers, and policymakers. It presents a well-structured study that moves logically from data collection to analysis and recommendations.

 The DH method is appropriate for assessing thermal discomfort, and the long-term data collection period (12 months) adds credibility to the findings. However *, *a brief explanation of the method might help readers unfamiliar with the concept.

 The paper provides significant insights into the thermal discomfort challenges of buildings in hot and humid climates, and its recommendations will be valuable for improving building design in such regions. Expanding on modern technologies and policy implications could further strengthen its impact: : although the paper provides practical recommendations, it could benefit from more detailed exploration of modern cooling technologies or innovations in building materials that could further alleviate discomfort.

Is the case presented with sufficient detail to be useful for teaching or other practitioners?

Yes

Is the work clearly and accurately presented and does it cite the current literature?

Yes

If applicable, is the statistical analysis and its interpretation appropriate?

Not applicable

Are all the source data underlying the results available to ensure full reproducibility?

Yes

Are the conclusions drawn adequately supported by the results?

Partly

Is the background of the case’s history and progression described in sufficient detail?

Yes

Reviewer Expertise:

Full professor, UNESCO Chair "Education and Research in Sustainable and Bioclimatic Urban Planning and Architecture", Civil Engineering, Building Energy Efficiency

I confirm that I have read this submission and believe that I have an appropriate level of expertise to confirm that it is of an acceptable scientific standard.
